# Phytoestrogens for Cancer Prevention and Treatment

**DOI:** 10.3390/biology9120427

**Published:** 2020-11-27

**Authors:** Margalida Torrens-Mas, Pilar Roca

**Affiliations:** 1 Grupo Multidisciplinar de Oncología Traslacional, Institut Universitari d’Investigació en Ciències de la Salut, Universitat de les Illes Balears, 07122 Palma, Spain; lida.torrens@uib.es; 2 Instituto de Investigación Sanitaria Illes Balears, 07010 Palma, Spain; 3 Ciber Fisiopatología Obesidad y Nutrición (CB06/03), Instituto Salud Carlos III, 28029 Madrid, Spain

**Keywords:** phytoestrogens, cancer prevention, cancer treatment, antioxidants

## Abstract

**Simple Summary:**

Phytoestrogens are compounds derived from plants that have a similar structure to human sex hormones. This has led to the observation that phytoestrogens have comparable effects to these hormones in our cells. Some beneficial effects of phytoestrogens include the improvement of menopausal symptoms and the prevention of several diseases. In fact, the consumption of soy and soy foods among the Asian population has been associated with a decrease in the incidence of some types of tumors. However, there are some concerns about whether these compounds may also have harmful effects, such as interfere with cancer treatments. In this review, we collect data on the reported effects of phytoestrogens alone or in combination with anti-cancer treatments and discuss the controversy around using these compounds.

**Abstract:**

Phytoestrogens are a large group of natural compounds found in more than 300 plants. They have a close structural similarity to estrogens, which allow them to bind to both estrogen receptors (ER), ERα and ERβ, presenting a weak estrogenic activity. Phytoestrogens have been described as antioxidant, anti-inflammatory, anti-thrombotic, anti-allergic, and anti-tumoral agents. Their role in cancer prevention has been well documented, although their impact on treatment efficiency is controversial. Several reports suggest that phytoestrogens may interfere with the effect of anti-cancer drugs through the regulation of oxidative stress and other mechanisms. Furthermore, some phytoestrogens could exert a protective effect on healthy cells, thus reducing the secondary effects of cancer treatment. In this review, we have studied the recent research in this area to find evidence for the role of phytoestrogens in cancer prevention and therapy efficacy.

## 1. Introduction

Phytoestrogens are naturally occurring compounds in plants and are characterized by a close structural similarity to estrogens. This allows them to act as weak estrogenic factors and interfere with hormonal signaling. Several reports suggest that phytoestrogens may have a positive effect on the prevention of menopausal symptoms, type 2 diabetes, cardiovascular disease, obesity, and cancer. These health benefits are presumably linked to their anti-inflammatory, anti-tumoral, anti-allergic, antioxidant, anti-thrombotic, and hepatoprotective properties [1]. The interest in phytoestrogens and cancer began after the observation that the consumption of soy and soy-derived foods was correlated with a decreased incidence of breast [2], ovarian [3], and prostate cancer [4]. In fact, the levels of genistein, the main soy isoflavonoid, and other phytoestrogens in plasma are inversely correlated to the risk of developing several types of cancer [5,6,7,8].

Phytoestrogens have been extensively tested in vitro and in vivo as anti-cancer treatments, although they have also been studied as adjuvant treatments to improve the response to chemotherapy, hormonotherapy, and radiotherapy. However, some studies alert that phytoestrogen consumption may interfere with cancer treatments and be harmful to patients. In this review, we analyze the current knowledge of the anti-tumoral properties of phytoestrogens and discuss their potential use as agents for cancer prevention and treatment. For this, we have searched the Pubmed (https://pubmed.ncbi.nlm.nih.gov/), Google Scholar (https://scholar.google.es/), and Scopus databases (https://www.scopus.com/home.uri) for articles regarding phytoestrogens and cancer from the last 25 years (1995–2020). To study the combination of phytoestrogens and anti-cancer treatments, especially the last six years (2014–2020) were considered. We also searched clinical trial databases to include those trials, finished or ongoing, using phytoestrogens for cancer patients.

## 2. Phytoestrogen Structure and Classification

**Figure 1:**
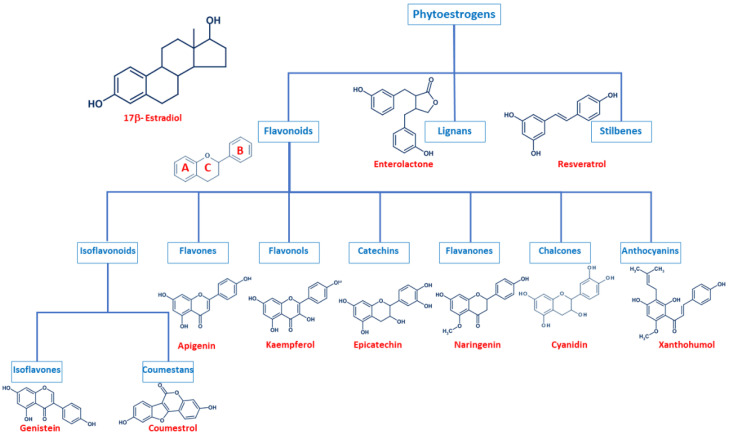
Comparison of the chemical structure of the different classes of phytoestrogens and 17β-estradiol.

Even though phytoestrogens are a large and heterogenic group, all of them are characterized by a phenolic ring and two hydroxyl groups (Figure 1), which are crucial for the binding to the estrogen receptors (ER). The agonist or antagonist properties of phytoestrogens depend on their phenolic group [1]. Based on their structure, phytoestrogens are classified into three main classes, which include flavonoids, lignans, and stilbenes [9,10].

### 2.1. Flavonoids

Flavonoids present the typical structure C6-C3-C6, with two aromatic rings (benzene A and B) joined together by a chain of 3 carbons cycled through an atom of oxygen (Figure 1) [11]. Flavonoids are commonly divided into several sub-classes, based on the connection position of the B and C rings, as well as the degree of saturation, oxidation, and hydroxylation of the C ring (Figure 1). This subclassification includes isoflavonoids (isoflavones and coumestans), flavones, flavonols, flavan-3-ols (or catechins), flavanones, chalcones, and anthocyanins [1,11,12,13,14].

Isoflavonoids are compounds derived from plant metabolism, and their structure consists of a 3-phenylchroman skeleton. They are also divided into two major groups, isoflavones, and coumestans. Isoflavones are flavonoids in which the B ring is linked to the heterocyclic ring at the C3 instead of the C2 position (Figure 1) [15]. Genistein and daidzein constitute up to 90% of isoflavones found in soybeans [16], and formononetin and biochanin A are mainly found in red clover. Isoflavones can be found in their free form or in their esterified forms [15]. Coumestans, the other subclass of isoflavonoids, have a 1-benzoxolo(3,2-c)chromen-6-one structure formed by a benzoxole fused with a chromen-2-one. One of the most studied coumestans is coumestrol, considered an endocrine disruptor, as it has the potential to bind to both ERs with similar affinity as estradiol, affecting the estrogenic signaling cascade [17]. Although the estrogenic activity of coumestrol is weaker than that of estradiol, it is 30 to 100 times greater than that of other isoflavones [18], due to the position of its two hydroxy groups, which match estradiol. This chemical structure also gives coumestrol the ability to inhibit aromatase and 3α-hydroxysteroid dehydrogenase [19], which are involved in the synthesis of steroid hormones [20].

Flavones are another type of flavonoids with a double bond between C2 and C3 (Figure 1). Furthermore, the C3 position does not have any substitution, and C4 is oxidized [21,22]. Luteolin and apigenin are the main compounds in this group. Due to its structure, luteolin is a strong inhibitor of xanthine oxidase, one of the main sources of ROS production [23]. On the other hand, apigenin is thought to protect cells against oxidative damage by enhancing mitochondrial function [23]. Furthermore, flavones may induce cell cycle arrest and DNA damage in some cell types, and specifically, apigenin may trigger apoptosis by inducing the activity of p38 kinase [24].

Flavonols are characterized by a 3-hydroxyflavone skeleton and are classified by the position of their phenolic group (Figure 1). Quercetin and kaempferol are the most predominant flavonols in plants [14]. Catechins or flavanols are mainly found in tea, vinegar, peach, and pome fruits. Epicatechin is an abundant polyphenol in unfermented cocoa beans, and it is thought to be responsible for the main health effects of cocoa. Another widely studied catechin is epigallocatechin gallate (EGCG), which is formed by the ester of epigallocatechin and gallic acid and is present in green tea. Both catechins have been associated with antioxidant and chemopreventive effects in several cell lines [25,26].

Flavanones are found in all citric fruits, and their chemical structure differs from flavones in the saturation of the C ring, with a saturated double bond between positions 2 and 3 (Figure 1) [12]. Naringenin is the most studied flavanone and contributes to limit lipid peroxidation and protein carbonylation by increasing antioxidant defenses [27]. Isoxanthohumol and 8-prenylnaringenin are also flavanones found in hop (*Humulus lupulus*), and they are also widely studied for their anti-cancer effects [28]. 8-prenylnaringenin has been identified as the most potent phytoestrogen and binds to both ERs [29], and inhibits aromatase [30]. Chalcones also belong to the flavonoids class and have a common 1,3-diaryl-2-propen-1-one skeleton, named chalconoid (Figure 1). The most studied chalcone is xanthohumol, also present in hop plants, and it possesses antibacterial and anti-cancer effects [31]. Finally, anthocyanins are the most abundant flavonoids in fruits and vegetables [32]. They are formed by a flavylium cation (2-phenylbenzopyrilium), which links hydroxyl (-OH) and/or methoxyl (-OCH3) groups. Various anthocyanins have been described, and mainly six are found in vegetables and fruits: pelargonidin, cyaniding, delphinidin, petunidin, peonidin, and malvidin [33].

### 2.2. Lignans

Lignans are another class of phytoestrogens commonly found in grains, nuts, coffee and tea, cocoa, flaxseed, and some fruits [34]. The chemical structure of lignans consists of two phenylpropane groups linked by a β-β’ bond, which is formed by a C-C bond between the central atoms of their side chains (position 8 or β) (Figure 1) [35,36,37,38]. Some studies report that these phytoestrogens are capable of mimicking the antioxidant effects of some hormones without any associated deleterious effects [35,39,40]. Importantly, gut bacteria are responsible for the metabolization of lignans and produce enterodiol and enterolactone. Thus, the beneficial health effects of lignans may be conditioned to each individual’s microbiota [41].

### 2.3. Stilbenes

Finally, stilbenes are an important group of nonflavonoid phytoestrogens with a polyphenolic structure with a 1,2-diphenylethylene nucleus (Figure 1) [42]. The most studied stilbene is resveratrol, a compound with two phenolic rings connected by a styrene double bond. This compound can occur in trans- and cis-isoforms, being the trans-isoform the most predominant one [43]. Resveratrol is found in a wide variety of dietary foods, including grapes, wine, nuts, and berries [44,45], and in fact, is considered a key compound in the French Paradox [46]. Several in vitro and in vivo studies report that resveratrol has anti-cancer properties, as well as antioxidant, anti-aging, anti-inflammatory and anti-pathogen effects [43,44,45,47,48].

## 3. Mechanism of Action of Phytoestrogens and Cancer Prevention

**Figure 2:**
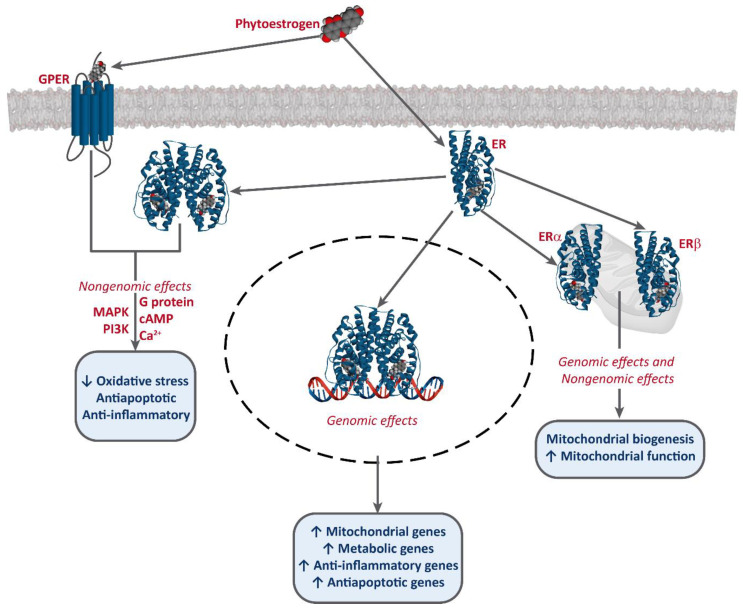
Interaction of phytoestrogens with ERs and GPER and their described effects.

It has been established that phytoestrogens interact with ERs, activating the transcription of several target genes. This results in the increase of the levels of antioxidants enzymes, such as superoxide dismutase (SOD), catalase (CAT), and glutathione peroxidase (Gpx), as well as an improvement of mitochondrial function [49,50,51]. Phytoestrogens also bind to G-protein-coupled estrogen receptor 1 (GPER/GPR30) and exert non-genomic effects [52,53,54] (Figure 2).

Phytoestrogens also have ER-independent effects. For instance, genistein and resveratrol can act as tyrosine kinase inhibitors, altering the activity of some downstream kinases [55]. Moreover, some studies also report an epigenetic mechanism for some phytoestrogens and the involvement of miRNA expression [56,57,58], and the modulation of chromatin structure [53,59]. However, they are mostly known as potent antioxidants, protecting cellular structures from ROS, such as epicatechin [25] or lignans [35], although the in vivo effects of these are much greater, due to the conversion into their active metabolites, enterolactone and enterodiol [60]. Furthermore, some studies report that at high concentrations, phytoestrogens may have an oxidant effect and induce cell death. This effect has been described for several compounds, including genistein [61,62,63,64], resveratrol [65,66], and xanthohumol [67,68,69]. Resveratrol is one of the most studied phytoestrogens, and several clinical trials have been developed to test its potential as a anti-cancer treatment [44,47,70], and some analogs are being designed [42,43].

These mechanisms of action have been associated with the chemoprevention potential of phytoestrogens. For instance, in Asian populations, soy consumption correlates with a lower incidence of prostate and breast cancer, which has been attributed to the presence of genistein in soy [71]. Genistein has a higher affinity for ERβ (87%) than for ERα (4%), and ERβ has been reported to have a protective effect against malignant transformation. In this regard, the chemoprevention effect of genistein may depend on the ERα/ERβ ratio [15,72,73,74].

Other phytoestrogens have been less widely studied, although they also show potential as chemopreventive agents. Quercetin may reduce the incidence of esophageal and stomach cancers, although this protection has not been observed for lung cancer [14,75,76,77]. There is also evidence for mild protection against gastrointestinal cancers associated with the consumption of tea flavanols [78]. Also, most epidemiological studies about lignans suggest that their intake reduces the risk of premenopausal breast cancer and possibly of postmenopausal breast cancer [39].

## 4. Phytoestrogens as Cancer Treatment

### 4.1. Phytoestrogens and Hormonal Therapy

The development and growth of some types of cancer are influenced by endocrine hormones, such as estrogens, progesterone, or androgens. Hormonal therapy is the main choice of treatment for these hormone-dependent cancers, which are breast, prostate, and uterine cancers [79]. This treatment consists of a specific modulator of the hormone receptor, which blocks its downstream signaling pathway. Tamoxifen (TAM) is one of the most commonly used drugs in hormonal therapy.

Controversial results are described for the combination of hormonotherapy with phytoestrogens. These different results could be explained, in part, because phytoestrogens may produce different effects in cancer cells depending on their Erα/ERβ ratio and the different affinity for these two receptors. Our group has reported that combining genistein and TAM increases the anti-tumor activity of this treatment in T47D cells (low ERα/ERβ ratio), while this combination decreases ROS production in MCF-7 cells (higher ERα/ERβ ratio) and increases cell viability [72]. Moreover, genistein seems to have an antagonizing effect on aromatase inhibitors for breast cancer at physiological concentration [80]. Constantinou et al. [81] reported that a diet combining daidzein and TAM results in increased protection from breast carcinogenesis in rats, although the combination with genistein antagonizes this chemopreventive effect. Tonetti et al. [82] also reported that genistein and daidzein antagonize TAM in vitro. On the other hand, a later study analyzing combining equol, a daidzein metabolite, and 4-hydroxy-tamoxifen, the bioactive metabolite of TAM, concluded that this strategy increases apoptosis and TAM efficacy in MCF-7 breast cancer cells [83]. The treatment with apigenin also shows the potential to overcome TAM resistance in SKOV3 ovarian cancer cells [84]. Interestingly, Zhang et al. [85] recently reported that rats that receive the combination treatment of TAM and genistein do not show any improvements; however, if the consumption of genistein starts at prepubertal or adult age, it shows a beneficial effect.

### 4.2. Phytoestrogens and Chemotherapy

Chemotherapy can be classified depending on the mechanism of action of each drug. This includes alkylating agents, anti-metabolites, topoisomerase inhibitors, mitotic inhibitors, and targeted therapies, among others. The development of resistance to these drugs is the main limitation of chemotherapy. The combination of chemotherapeutic agents with phytoestrogens has been studied to overcome resistance to treatment. There are controversial results, and very few clinical studies have been fully developed to this date, since most of them have been canceled, due to their lack of effectiveness.

Alkylating agents include platin-based therapies, such as cisplatin and oxaliplatin, and other drugs, such as dacarbazine. These agents form adducts in DNA and interfere with DNA repair mechanisms, eventually stopping cells from dividing. They are used to treat blood cancers, sarcomas, and lung, bladder, breast, and ovarian cancers [86]. The effects of combining phytoestrogens and alkylating agents seem to be dependent on the type of cancer. Genistein may help to overcome resistance to cisplatin in gastric cancer [87] and could contribute to reducing the dose of cisplatin used for BxPC-3 pancreatic cancer cell line by blocking the cisplatin-induced activation of NF-κB [88]. However, other studies report that genistein and daidzein show an antagonizing effect in combination with cisplatin in medulloblastoma, breast, and colon cancer cells [89,90].

Other phytoestrogens also show the potential to increase the efficacy of alkylating agents in several studies. The combination of cisplatin with apigenin seems to inhibit cell proliferation in breast cancer cell lines and decreases telomerase activity, limiting one of the mechanisms to escape apoptosis and induce metastasis [91]. Another study found that apigenin increases the efficacy of cisplatin in B16-BL6 melanoma cells in vivo, significantly reducing tumor volume in mice [92]. Resveratrol, in combination with cisplatin in SW620 and HT-29 colon cancer cells, results in lower cell viability compared to the treatment alone [93], while the combination with oxaliplatin increased its anti-tumor activity in SW480 and SW620 cells [94]. Quercetin also improves the response of lung cancer cells to cisplatin, although this effect was not attributed to an increase in antioxidant enzymes [95]. On the other hand, Sharma et al. [96] reported that this combination increased oxidative stress and cytotoxicity in HeP2 laryngeal carcinoma cells. On the contrary, for ovarian cancer, only the combination of cisplatin and kaempferol had success in overcoming cisplatin resistance in OVCAR-3 cells, while apigenin, genistein, and quercetin showed no improvement [97]. EGCG may also potentiate the anti-tumor activity of cisplatin in ovarian cancer cells [98].

Some phytoestrogens do not have any influence on the activity of cisplatin, but show other beneficial effects. For instance, cotreatment of formononetin and cisplatin inhibits apoptosis in kidney epithelial cells by suppressing ROS production, suggesting a protective effect against secondary effects [99]. Furthermore, treatment with biochanin A before cisplatin treatment triggers the activation of the Nrf2 pathway, resulting in a protective effect against nephrotoxicity, which is a common complication of cisplatin treatment [100].

Mitotic inhibitors usually act by binding to tubulin or by inducing microtubule disassembly, preventing mitosis. These drugs include docetaxel, paclitaxel, or vincristine, and are used to treat breast, lung, and blood cancers [86]. In B-cell tumors, the cotreatment of resveratrol and paclitaxel in vitro synergistically increases apoptosis, suggesting a sensitizing effect that could reduce the dose of paclitaxel [101]. Öztürk et al. [102] also showed this synergistic effect in glioblastoma cancer cells, which increased ROS production and induced apoptosis. EGCG also increases the efficacy of docetaxel and paclitaxel in prostate cancer cells [103] and the activity of paclitaxel in breast cancer cells and in vivo [104]. Another study showed that isoxanthohumol may also be a potential coadjuvant for melanoma, as it improves the anti-cancer activity of paclitaxel both in vivo and in vitro [105]. A pretreatment of genistein for 24 h also shows a sensitizing effect on docetaxel in BxPC-3 pancreatic cancer cell line, inhibiting NF-κB signaling, and triggering apoptosis [88].

Anti-metabolites are commonly used drugs to treat leukemias and solid tumors, such as breast, ovarian, and intestinal tract cancers. The most used are 5-fluorouracil (5-FU) and gemcitabine, and they interfere with DNA and RNA synthesis, as they substitute the usual metabolites [86]. Several reports have studied resveratrol in combination with 5-FU. Our group has previously reported that treatment with resveratrol in combination with 5-FU increases oxidative stress in colon cancer cells and shows higher cytotoxicity compared to the 5-FU alone [65,93]. Moreover, combining resveratrol with 5-FU is also effective in a model on murine liver cancer, allowing a reduction of the administered dose of 5-FU [106], and in B16 melanoma cells in vitro and in vivo [107]. Resveratrol can also sensitize pancreatic cancer cells to gemcitabine in vitro and in vivo [108]. Frampton et al. [109] also reported that resveratrol increases the anti-tumor effect of both 5-FU and gemcitabine in cholangiocarcinoma cell lines.

Genistein has been shown to increase the cytotoxic effect of gemcitabine in different cancer types, presumably overcoming chemoresistance by suppressing the Akt/NF-κB pathway induced by chemotherapy, as shown in osteosarcoma cells [110], ovarian cancer cells [111], and in a mouse model of pancreatic cancer [112]. Genistein has also shown the potential to synergize with 5-FU in pancreatic cancer cells and in in vivo models [113]. Finally, Tang et al. [114] reported that EGCG also increases the activity of gemcitabine in pancreatic cancer cells.

Topoisomerases are the enzymes responsible for the uncoiling of DNA during DNA replication. Irinotecan, topotecan, and etoposide are the most used topoisomerase inhibitors in clinical practice to treat lung, ovarian and gastrointestinal cancers, as well as some leukemias [86]. Several studies have reported an improvement of the cytotoxic activity of these drugs in combination with phytoestrogens. For instance, a combination of etoposide and resveratrol results in lower cell viability in SW620 and HT-29 colon cancer cells compared to the chemotherapy treatment alone [93]. Guo et al. [115] recently showed that the combination of daidzein with topotecan results in lower cell viability both in vitro and in vivo, as well as a reversal of resistance to chemotherapy.

Anti-tumor antibiotics act by interfering with the enzymes involved in DNA replication or causing strand breakage. The most common include daunomycin, doxorubicin, and epirubicin. These drugs are commonly used to treat soft tissue sarcomas and hematological cancers, as well as some types of carcinoma, although a major setback of these drugs is their cardiotoxicity [86]. Doxorubicin is probably the most studied drug in combination with phytoestrogens, and contradictory results have been obtained. Rigalli et al. [116] showed that treatment of genistein in MCF-7 and MDA-MB-231 breast cancer cell lines increases their chemoresistance to doxorubicin. Another study showed that this combination does not improve the cytotoxic activity of doxorubicin in MCF-7 cells [117]. However, Xue et al. [118] reported that genistein-treated cells overcome their resistance to doxorubicin.

The combination of EGCG with doxorubicin produces synergistic effects inhibiting metastasis and cell proliferation in prostate cancer cells and in vivo [119]. Reedijk et al. [120] also showed that catechins may increase the anti-tumor activity of doxorubicin in hepatocarcinoma cell lines and in a mice model. Du et al. [121] showed that luteolin increases the efficacy of doxorubicin and decreases its secondary effects by increasing the antioxidant capacity of serum, while Staedler et al. [122] reported the same results with the combination of doxorubicin and quercetin. The combination of kaempferol with doxorubicin increases ROS production and cell death, increasing the efficacy of the drug in glioblastoma cells [123]. In acute myeloid leukemia cells resistant to doxorubicin, treatment with resveratrol for 24 h seems to overcome drug resistance and increase apoptosis [124]. Resveratrol has also been described to protect from doxorubicin-induced cardiotoxicity [45]. Formononetin also increases the sensitivity to doxorubicin in some glioma cell lines, although in this case, the antioxidant activity was not tested [125]. The combination of doxorubicin with biochanin A also results in a synergistic effect inhibiting cell proliferation of osteosarcoma cells [126].

Dash and Konkimalla [127] recently reported that the encapsulation in liposomes of biochanin A and doxorubicin increases the uptake of chemotherapy in COLO205 doxorubicin-resistant colon cancer cells and increases their sensitivity to the drug. The encapsulation of genistein and doxorubicin also shows potential for metastatic prostate cancer [128].

Exposure of Caco-2 cells to 8-hydroxydaidzein, a daidzein derivative that is produced in the liver, increases ROS levels and the sensitivity of these cells to epirubicin, as evidenced by the p53-induced cell cycle arrest and triggering of apoptosis [129]. Somjen et al. [130] performed an in vivo study where they conjugated another derivative of daidzein with daunomycin, improving the efficiency of the drug in ovarian cancer, and showed less secondary effects, such as weight loss in mice. Trichostatin A (TSA) is also an antibiotic used as chemotherapy for prostate, breast, and gastrointestinal cancer. This compound can inhibit histone deacetylases in mammalian cells. The combination of TSA with genistein significantly reduces the viability of Hep-2 laryngeal cancer cells [131] and A549 lung cancer cells [132].

Finally, targeted therapies include small molecules and antibodies that target some proteins or block signaling pathways in cancer cells. The most studied include sunitinib, bortezomib, and sorafenib [133]. Genistein seems to contribute to enhancing the effect of sorafenib in hepatocellular carcinoma in vivo [134]. On the contrary, Rigalli et al. [135] reported that genistein, at concentrations of 1 and 10 µM, induces a higher resistance of HepG2 hepatocellular carcinoma cells to sorafenib.

Resveratrol is also a promising adjuvant for this type of therapy. Ivanova et al. [136] recently showed that combining resveratrol with some targeted drugs, such as barasertib, synergistically increases ROS production and apoptosis in leukemic cells, while leaving normal lymphocytes unaltered. Moreover, resveratrol also improves the efficacy of sorafenib in MCF-7 breast cancer cells by increasing ROS production and apoptosis [137]. The combination of sorafenib and biochanin A in some hepatocarcinoma cell lines also results in synergistic effects increasing cell death [138], and formononetin has been reported to increase the cytotoxic effect of sunitinib in vivo [139]. Finally, navitoclax (ABT-263) is an experimental drug currently in evaluation for solid tumors and non-Hodgkin’s lymphoma. The combination of Navitoclax and apigenin synergistically enhanced the effect of this drug in several colon cancer cell lines and suppressed tumor growth by 70% in xenograft mice, without observing a worsening of secondary effects [140].

Some anti-cancer compounds can be classified into prodrugs. These molecules have little or no effect until they are metabolized and converted into their active form. This strategy is often used to overcome problems with drug bioavailability, and more recently, with tissue-selective distribution [141,142]. Phytoestrogens themselves have been considered as prodrugs. Some examples are lignans, which, as mentioned before, are metabolized into their active compounds by gut bacteria; daidzein and formononetin, metabolized into equol also by intestinal bacteria; apigenin and kaempferol, converted into luteolin and quercetin [143,144], respectively, and activation of chalcones by the action of the cytochrome P450 enzyme [145]. Several strategies to develop prodrugs of phytoestrogens are currently under study and are reviewed elsewhere [146,147]. Furthermore, some studies have analyzed the combination of phytoestrogens with other prodrugs. For instance, the combination of genistein and a prodrug of vitamin D synergistically decreases the proliferation of prostate and breast cancer cells in vitro [148]. Daidzein has also been used in combination with a prodrug based on allicin, to specifically target ovarian cancer cells in an animal model [149]. Di et al. [150] showed that a combination of a specific antibody targeting prostate cancer cells, glucuronidase, and enterolactone glucuronide could decrease the dose of docetaxel used. Finally, another study developed a new potential prodrug based on benzimidazole combined with chalcones with comparable or higher effectivity than cisplatin in breast and ovarian cancer cells in vitro [151].

Even though the combination of phytoestrogens and chemotherapy shows some promising effects in vitro and in some in vivo models, the clinical studies that have been carried out reported little significant improvements. For instance, a phase II study testing the addition of genistein to gemcitabine and erlotinib treatments for pancreatic cancer patients reported no improvement in the anti-tumor activity of these drugs, although the combination was well tolerated [152]. Another phase I clinical study showed that the combination of a derivative of genistein and gemcitabine may be beneficial for some specific pancreatic cancer patients [153]. This may open the door to the development of drugs based on the structure of phytoestrogens with some modifications to make them potential coadjuvant treatments.

### 4.3. Phytoestrogens and Radiotherapy

Ionizing γ-radiation is the major choice for radiotherapy, although it has adverse effects, such as immunosuppression, inflammation, epigenetic modulation, necrosis, and secondary carcinogenesis induction, among others. These secondary effects are mainly due to the induction of ROS production, which leads to cellular oxidative damage [154]. Since phytoestrogens have antioxidant properties, they have been studied as radioprotective compounds for non-tumor cells. However, other studies support that high doses of some phytoestrogens may act as pro-oxidant molecules, thus increasing the sensitivity of cancer cells to radiotherapy. The most studied phytoestrogens as radiosensitizers and radioprotectors are genistein and resveratrol, although there are reports on the effect of other compounds.

In HL-60 leukemia cells, treatment with genistein induces cell cycle arrest and ROS production, which renders cells more sensitive to γ-radiation. At the same time, genistein has a protective effect against radiation on normal lymphocytes [155]. This radiosensitizing effect was also reported for prostate cancer cells and in in vivo experiments [156]. Genistein and daidzein also show potential as radiosensitizers in PC-3 prostate cancer cells in vitro and in vivo [157]. Resveratrol has been described to improve sensitivity in melanoma cells [158], prostate cancer cells [159], and cervical cancer cells [160,161]. Finally, naringin, a glycone form of naringenin usually found in citrus fruits, has shown potential as a radioprotector in mice, particularly in the spleen, one of the most sensitive organs to radiation. A pretreatment with naringin may protect cells from radiation-induced oxidative damage by increasing the expression and activity of antioxidant enzymes and suppressing the activation of NF-κB, thus attenuating the adverse effects of radiotherapy [162].

Table 1 summarizes all the positive, neutral, and negative effects described for the combination of phytoestrogens and different choices of anti-cancer treatment.

## 5. Conclusions

**Figure 3:**
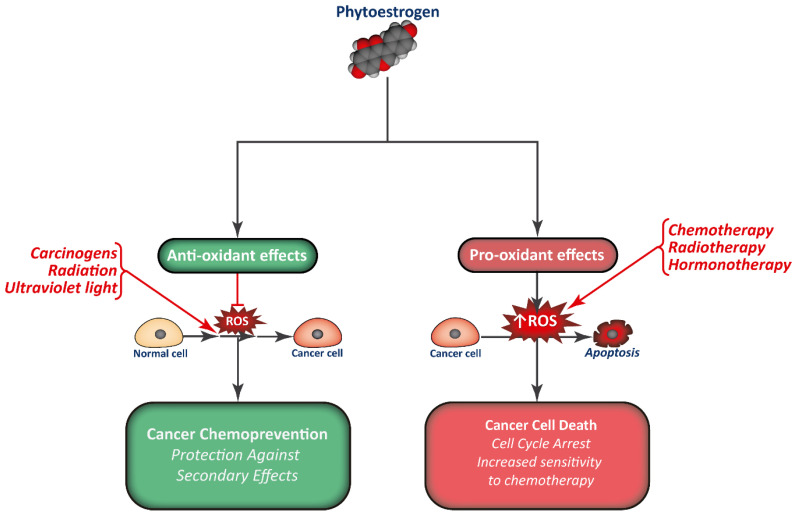
Combination of phytoestrogens with anti-cancer treatments may render cancer cells more sensitive to treatment, in part by increasing ROS production, while normal cells may be protected from the secondary effects of therapy.

Phytoestrogen consumption has been associated with a reduction in cancer incidence, and they are studied as promising chemopreventive compounds. Apart from interfering with the normal signaling pathways of estrogens and modulate gene expression, phytoestrogens are also potent antioxidants, modulate normal protein activity, and regulate epigenetics (Figure 3). This way, phytoestrogens have the potential to limit cell proliferation in different types of tumors.

Furthermore, phytoestrogens may sensitize cancer cells to anti-cancer treatments, including hormonotherapy, chemotherapy, and radiotherapy. Some reports also show that phytoestrogens could also protect normal cells from the secondary effects without affecting the efficacy of treatment. However, further research and clinical studies must be carried out to evaluate the true potential of phytoestrogens as an option for cancer therapy, establish the optimal concentration and which patients could benefit from it, and ensure their safety. Until now, most clinical studies regarding phytoestrogens and cancer have been canceled, due to a lack of effect. In this regard, several investigations are focused on designing analogs or strategies, such as encapsulation, to improve the efficacy of phytoestrogen as treatments or coadjuvants for some types of cancer.

## Figures and Tables

**Table 1:** Summary of the described effects of phytoestrogens in combination with anti-cancer therapies.

Phytoestrogen	Treatment Combination	Cancer Type	Effect	Reference
Apigenin	Tamoxifen	Ovarian Cancer	+	[84]
	Ciaplatin	Breast and Ovarian Cancer, Melanoma	+/N.E.	[91,92,97]
	Navitoclax	Colon Cancer	+	[140]
Biochanin A	Doxorubicin	Osteosarcoma, Colon Cancer	+	[126,127]
	Sorafenib	Hepatocarcinoma	+	[138]
	Cisplatin	Kidney Cells	S.E. improvement	[99,100]
Catechins	Doxorubicin	Hepatocarcinoma	+	[120]
Daidzein	TAM	Breast Cancer	−	[82]
	Cisplatin	Medulloblastoma, Breast, and Colon Cancer	−	[89,90]
	Topotecan	Breast Cancer	+	[115]
	Epirubicin	Colon Cancer	+	[129]
	Daunomycin	Ovarian Cancer	+, S.E. improvement	[130]
	Radio	Prostate Cancer	+	[157]
EGCG	Oxaliplatin	Ovarian Cancer	+	[98]
	Docetaxel, paclitaxel	Prostate and Breast Cancer	+	[103,104]
	Gemcitabine	Pancreatic Cancer	+	[114]
	Doxorubicin	Prostate Cancer	+	[119]
Equol	TAM	Breast Cancer	+	[83]
Formononetin	Sunitibib	Breast Cancer	+	[139]
	Doxorubicin	Glioma	+	[125]
	Cisplatin	Kidney Cells	S.E. improvement	[99,100]
Genistein	Tamoxifen	Breast Cancer	+/−	[72]
	Fadrozole (Aromatase Inhibitor)	Breast Cancer	−	[80]
	Tamoxifen	Breast Cancer	+/−	[81,82]
	Cisplatin	Gastric and pancreatic Cancer	+	[87,88]
	Docetaxel	Pancreatic Cancer	+	[88]
	Cisplatin	Medulloblastoma, Breast, Ovarian, and Colon Cancer	−/N.E.	[89,90,97]
	Gemcitabine	Osteosarcoma, Ovarian and Pancreatic Cancer	+	[110,111,112]
	5-FU	Pancreatic Cancer	+	[113]
	Doxorubicin	Breast Cancer	−/+/N.E.	[116,117,118]
	Doxorubicin	Prostate Cancer	+	[128]
	TSA	Lung and Laryngeal Carcinoma	+	[131,132]
	Sorafenib	Hepatocarcinoma	+/−	[134,135]
	Radio	Leukemia and Prostate Cancer	+, S.E. improvement	[155,156,157]
Isoxanthohumol	Paclitaxel	Melanoma	+	[105]
Kaempferol	Cisplatin	Ovarian Cancer	+	[97]
Kaempferol	Doxorubicin	Glioblastoma	+	[123]
Luteolin	Doxorubicin	Breast Cancer	+	[121]
Naringin	Radiotherapy	Splenocytes	S.E. improvement	[162]
Quercetin	Doxorubicin	Breast Cancer	+	[122]
	Cisplatin	Ovarian Cancer, Lung, and Laryngeal Carcinoma	+	[95,96,97]
Resveratrol	Cisplatin	Colon Cancer	+	[93]
	Etoposide	Colon Cancer	+	[93]
	Oxaliplatin	Colon Cancer	+	[94]
	5FU	Colon and Liver Cancer, Melanoma, and Cholangiocarcinoma	+	[65,93,106,107,109]
	Paclitaxel	B-cell Malignancies, Glioblastoma	+	[101,102]
	Gemcitabine	Pancreatic Cancer and Cholangiocarcinoma	+	[108,109]
	Doxorubicin	Acute Myeloid Leukemia	+, S.E. improvement	[45,124]
	Barasertib	Leukemia	+	[136]
	Sorafenib	Breast Cancer	+	[137]
	Radiotherapy	Melanoma, Prostate, and Cervical Cancer	+	[158,159,160,161]

Phytoestrogens are ordered alphabetically. + indicates an increase in efficacy of the anti-cancer treatment; − indicates interference with the anti-cancer treatments; N.E., no effects; S.E. improvement, side effects improvement.
